# Seropositivity for Anti-HCV Core Antigen is Independently Associated With Increased All-Cause, Cardiovascular, and Liver Disease-Related Mortality in Hemodialysis Patients

**DOI:** 10.2188/jea.JE20100187

**Published:** 2011-11-05

**Authors:** Masaki Ohsawa, Karen Kato, Kozo Tanno, Kazuyoshi Itai, Yosuke Fujishima, Akira Okayama, Tanvir Chowdhury Turin, Toshiyuki Onoda, Kazuyuki Suzuki, Motoyuki Nakamura, Kazuko Kawamura, Takashi Akiba, Kiyomi Sakata, Tomoaki Fujioka

**Affiliations:** 1 Department of Hygiene and Preventive Medicine, Iwate Medical University, Iwate, Japan; 2 Division of Urology, San-ai Hospital, Iwate, Japan; 3 Department of Urology, Iwate Medical University, Morioka, Japan; 4 The First Institute of Health Service, Japan Anti-Tuberculosis Association, Tokyo, Japan; 5 Department of Medicine, University of Calgary, Calgary, Alberta, Canada; 6 Division of Gastroenterology and Hepatology, Department of Internal Medicine, Iwate Medical University, Morioka, Japan; 7 Division of Cardiovascular Medicine, Nephrology and Endocrinology, Department of Internal Medicine, Iwate Medical University, Morioka, Japan; 8 Iwate Health Service Association, Morioka, Japan; 9 Division of Blood Purification, Kidney Center, Tokyo Women’s Medical University, Tokyo, Japan

**Keywords:** hepatitis C virus, hemodialysis, mortality, population-based cohort study

## Abstract

**Background:**

It is not known whether chronic or past hepatitis C virus (HCV) infection contributes to the high mortality rate in hemodialysis patients.

**Methods:**

This prospective study of 1077 adult hemodialysis patients without hepatitis B virus infection used Poisson regression analysis to estimate crude and sex- and age-adjusted rates (per 1000 patient-years) of all-cause, cardiovascular, infectious disease-related and liver disease-related mortality in patients negative for HCV antibody (group A), patients positive for HCV antibody and negative for anti-HCV core antigen (group B), and patients positive for anti-HCV core antigen (group C). The relative risks (RRs) for each cause of death in group B vs group C as compared with those in group A were also estimated by Poisson regression analysis after multivariate adjustment.

**Results:**

A total of 407 patients died during the 5-year observation period. The sex- and age-adjusted mortality rate was 71.9 in group A, 80.4 in group B, and 156 in group C. The RRs (95% CI) for death in group B vs group C were 1.23 (0.72 to 2.12) vs 1.60 (1.13 to 2.28) for all-cause death, 0.75 (0.28 to 2.02) vs 1.64 (0.98 to 2.73) for cardiovascular death, 1.64 (0.65 to 4.15) vs 1.58 (0.81 to 3.07) for infectious disease-related death, and 15.3 (1.26 to 186) vs 28.8 (3.75 to 221) for liver disease-related death, respectively.

**Conclusions:**

Anti-HCV core antigen seropositivity independently contributes to elevated risks of all-cause and cause-specific death. Chronic HCV infection, but not past HCV infection, is a risk for death among hemodialysis patients.

## INTRODUCTION

Hepatitis C virus (HCV) infection, currently the most common blood-borne infection, is an emerging public health problem.^1^ Only 20% to 30% of patients with acute HCV infection spontaneously recover; the rest develop chronic HCV infection. Most patients who recover from HCV infection do not develop liver cirrhosis or hepatocellular carcinoma (HCC), whereas patients with chronic HCV infection develop liver cirrhosis or HCC within 20 to 30 years of initial infection.^2^

Hemodialysis patients are especially vulnerable to HCV infection, because of exposure associated with dialysis and blood transfusion.^3^^–^^5^ The prevalence of HCV in hemodialysis patients is very high (2.7%–30.0%).^6^^–^^21^ Studies suggest that HCV infection independently contributes to increased mortality among hemodialysis patients.^22^^–^^27^ However, it is not known whether chronic HCV infection or a history of past HCV infection increases mortality. Moreover, it has not been established whether the elevated mortality risk due to HCV infection is mostly attributable to an increase in liver disease-related deaths.

To assess the contribution of past and chronic HCV infection among hemodialysis patients, we estimated the relative risks of all-cause and cause-specific death attributable to HCV antibody seropositivity and anti-HCV core antigen seropositivity.

## METHODS

### Participants

**Figure 1.**
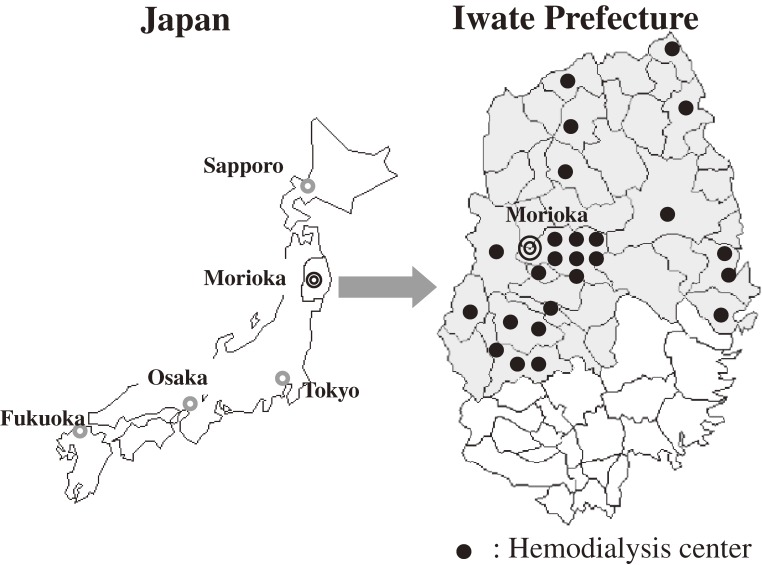
Map of the KAREN Study area. A map of Japan. Morioka, the capital of Iwate Prefecture, is located in northeast Honshu island. The KAREN Study area (the shaded area covering about two-thirds of Iwate Prefecture) has 26 hemodialysis centers. Only 1 center, which treats 7 patients, was not included in the study. Each closed circle represents a hemodialysis center.

**Figure 2.**
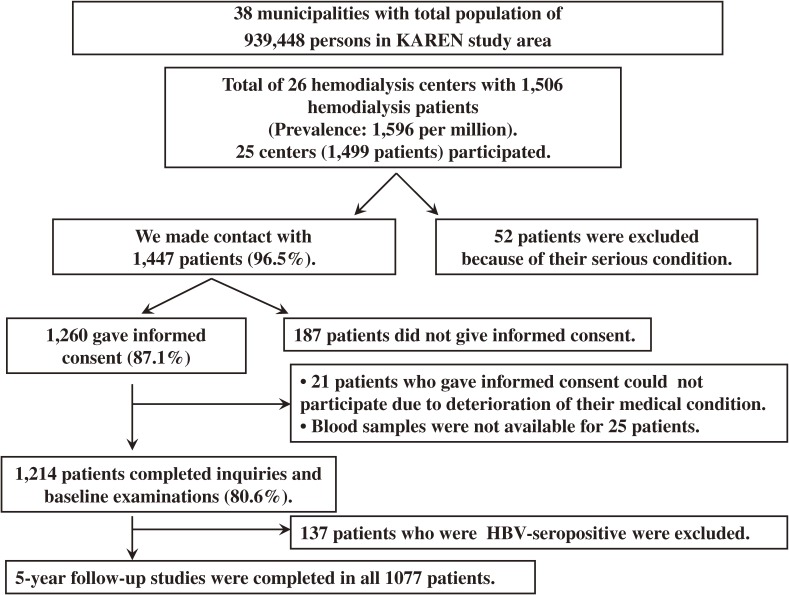
Flow chart of the procedure used to select patients for participation in the KAREN Study. There were 1506 adults receiving hemodialysis in 26 centers in the study area. We were able to contact 1447 patients (96.5%). Fifty-two patients were excluded because of their serious clinical condition. A total of 1260 patients (87.1%) provided written informed consent for participation in the study, and 1214 patients (80.6%) completed the baseline examinations. A total of 137 patients who were positive for hepatitis B surface antigen were excluded. Finally, data from 1077 patients were analyzed. We ascertained the vital status of all participants after completion of a 5-year follow-up survey.

The eligible participants were adult hemodialysis patients who participated in the KAREN study, a population-based prospective study that has been conducted since 2003 in northern Japan (Figure 1).^28^ A total of 1214 adult hemodialysis patients (80.6% of all hemodialysis patients in the study area; age 22 to 95 years; 779 men and 435 women) are included in the KAREN study. The participants in the KAREN study are patients who were undergoing adult hemodialysis in April 2003. A total of 137 patients who were positive for hepatitis B surface antigen were excluded. Ultimately, data from 1077 patients were analyzed. We ascertained the vital status of all subjects in a 5-year follow-up survey (Figure 2). All participants gave written informed consent to participate. This study was approved by the Medical Ethics Committee of Iwate Medical University and conducted in accordance with the guidelines of the Declaration of Helsinki.

### Data collection

The initial investigations in the KAREN Study consisted of a questionnaire, review of medical records, measurements of blood pressure and anthropometric data, and blood testing. The data gathering methodology was previously described.^21^^,^^28^ Information on HCV antibody serology testing was collected by reviewing medical charts.^21^

Results of anti-HCV antibody tests could not be obtained from chart review for 50 patients. Frozen serum samples from those patients were thawed and anti-HCV antibody tests were performed using a second-generation assay (Architect HCV, Abbott Laboratories, Japan). Frozen samples from patients who were positive for anti-HCV antibody were thawed, and HCV core antigen tests were performed using a chemiluminescent enzyme immunoassay (Lumispot Eiken HCV antigen, Eiken Chemical Co., LTD, Japan).^21^

### Outcomes

Follow-up studies were performed annually at each center. Members of the KAREN Study team reviewed all the medical records of study participants. The medical records of deceased patients were summarized. Cause of death was independently determined by physicians on the KAREN Outcome Review Committee, based on the summaries. Disagreements regarding cause of death were discussed, and the final determination was reached by consensus. We identified the 3 major causes of death (cardiovascular, infectious disease-related, and liver disease-related) using codes form the Tenth Revision of the International Classification of Diseases (ICD-10; Table 1).

**Table 1.** Criteria for determining causes of death in the KAREN Study (based on ICD-10)

Cardiovascular death: I01–I99 plus R96			
	cardiac death: I20–I25, I27, I29, I30–I52		
		I20–I25	coronary artery disease
		I33	Acute and subacute endocarditis
		I50	heart failure
	pulmonary embolism: I26		
	stroke death: I60–I69		
		I60	subarachnoidal hemorrhage
		I61, I62	intracerebral hemorrhage
		I63	cerebral infarction
		I64, I67	other type of stroke
	vascular death: I70–I77		
		I70	Atherosclerosis
		I71	aortic anuerysm and dissection
		I72, I73	other peripheral artery disease
		I74	arterial embolism and thrombosis
		I77	other arterial disease
	sudden cardiac death: I46, I49, R96		
	cardiac arrest: I46		
		I46.0	Cardiac arrest with successful resuscitation
		I46.1	Sudden cardiac death, so described
		I46.9	Cardiac arrest, unspecified
	ventricular fibrillation and flutter: I49		
		I49.0	Ventricular fibrillation and flutter
	other sudden death, cause unknown R96		
		R96.0	Instantaneous death
		R96.1	Death occurring less than 24 hours from onset of symptoms, not otherwise explained
Infectious disease-related death:			
	A: bacterial infection: A00–A09, A15–A19, A40–A41 (septicemia)		
	B: viral infection, fungal and other microorganism infection		
	G: infectious diseases in nervous system		
		G00	Bacterial meningitis, not elsewhere classified
		G04.2	Bacterial meningoencephalitis and meningomyelitis, not elsewhere classified
	J: infectious diseases in respiratory tract		
		J10–J11	influenza
		J12–J18	pneumonia
		J20	acute bronchitis
		J69	Pneumonitis due to solids and liquids
		J86	Pyothorax
	K: infectious diseases in gastrointestinal tract and digestive organ		
		K65	Peritonitis
		K80.3	Calculus of bile duct with cholangitis
		K81	Cholecystitis
	L: infectious diseases in skin and subcutaneous tissue		
		L03	Cellulitis
		L89	Decubitus ulcer
Liver disease-related death			
		K71.2	Toxic liver disease with acute hepatitis
		K72	Acute and subacute hepatic failure
		K73	Chronic hepatitis, not elsewhere classified
		K74	Fibrosis and cirrhosis of liver
		C22.0	Liver cell carcinoma

### Classification and definition

The participants were divided into 3 groups based on the results of HCV antibody testing and anti-HCV core antigen testing at the baseline survey. Group A consisted of patients who were negative for anti-HCV antibodies (*n* = 968). Group B consisted of patients who were positive for anti-HCV antibodies and negative for anti-HCV core antigen antibodies (*n* = 55). Group C consisted of patients who were positive for both anti-HCV antibodies and anti-HCV core antigen antibodies (*n* = 79). These 3 groups were selected because they roughly corresponded to patients without HCV infection (group A), patients with past HCV infection (group B), and patients with chronic HCV infection (group C).^21^

High blood pressure was defined as systolic blood pressure (SBP) in the highest quartile of this study population (SBP ≥169 mm Hg). Low blood pressure was defined as SBP in the lowest quartile (<140 mm Hg). Diabetes was defined as a nonfasting plasma glucose level of 200 mg/dL or higher, a plasma HbA1c of 6.5% or higher, use of antidiabetic medication, or a combination thereof. Dyslipidemia was defined as serum total cholesterol (TC) of 220 mg/dL or higher, serum high-density lipoprotein cholesterol (HDL-C) level less than 40 mg/dL, use of antidyslipidemic medication, or a combination thereof. High body mass index (BMI) was defined as a BMI of 27.5 kg/m^2^ or higher. Low BMI was defined as a BMI less than 18.5 kg/m^2^. High-sensitivity C reactive protein (hs-CRP) level was considered high if it was in the highest quartile (≥3.6 mg/L). Hypoalbuminemia was defined as a serum albumin level less than 3.5 mg/dL. A smoking habit was defined as current smoking. Regular drinking was defined as alcohol consumption on 5 or more days per week.

### Statistical analysis

Risk factor-related variables were expressed as sex- and age-adjusted means plus 95% CI and compared across HCV infection status groups using analysis of covariance (ANCOVA). The hs-CRP level was expressed as a sex- and age-adjusted geometric mean plus 95% CI. The χ^2^ test was used to compare frequencies.

We defined the follow-up period as the period from the initial survey to the first outcome or the end of observation. Individuals who were free of outcomes in the 5-year follow-up study were administratively censored. The cumulative probability of each cause of death was estimated using the Kaplan-Meier method, and differences in the cumulative probability of death were assessed by the log-rank test. Crude mortality rates and sex- and age-adjusted mortality rates were estimated in the 3 groups (groups A, B, and C) by Poisson regression analysis in which multivariate-adjusted mortality rate ratios and their 95% CIs were calculated in groups B and C, with those of group A serving as reference. The variables used in the multivariate adjustment were traditional risk factors, including age, male sex, high BMI, dyslipidemia, diabetes, high blood pressure, history of myocardial infarction, stroke, or malignant disease, smoking habit, and regular drinking habit (model A). Hemodialysis-related risk factors, including low BMI, low blood pressure, high CRP level, and hypoalbuminemia, were also additionally used as explanatory variables in model B. All *P* values were 2-tailed, and values less than 0.05 were considered to indicate statistical significance. The statistical package PASW (version 18.0, IBM Japan Inc., Tokyo, Japan) was used for the statistical analysis.

## RESULTS

Table 2 shows the baseline characteristics of the patients, stratified by HCV infection status. The proportions of patients in groups A, B, and C were 90.0%, 3.6%, and 6.5%, respectively. As compared with patients in group A, those in group C had significantly lower serum TC, serum low-density lipoprotein cholesterol (LDL-C), serum albumin, and serum creatinine levels, and lower platelet and white blood cell (WBC) counts (*P* < 0.05 for all tests). Patients in group B had significantly lower systolic blood pressure, TC, and LDL-C levels, and lower platelet and WBC counts than did patients in group A (*P* < 0.05 for all tests).

**Table 2.** Baseline characteristics of patients stratified by HCV seropositivity

HCV seropositivity status groups (number of subjects)	group A HCV Ab(−) *n* = 968	group B HCV Ab(+) Ag(−) *n* = 39	group C HCV Ab(+) Ag(+) *n* = 70
male *n*, (%)	605 (62.5%)	25 (64.1%)	53 (75.7%)
mean age (SD) (yrs)	61.2 (13.3)	61.1 (13.6)	58.8 (10.9)
median vintage of HD (25–75%) (yrs)	4.5 (1.9–8.3)	8.9 (3.6–21.2)	8.3 (2.4–21.8)

Sex- and age-adjusted mean levels and their 95% CIs of anthropometrical and blood test measurements			
body mass index (kg/m^2^)	21.0 (20.8–21.2)	20.6 (19.6–21.5)	20.2 (19.5–20.9)
SBP (mm Hg)	156 (154–157)	146 (138–153)^b^	155 (149–161)
total cholesterol level (mg/dl)	157 (155–159)	140 (129–150)^c^	136 (128–144)^c^
HDLC (mg/dl)	47.1 (46.1–48.0)	43.4 (38.7–48.1)	44.7 (41.2–48.2)
LDLC (mg/dl)	86.1 (84.4–87.8)	74.2 (65.9–82.5)^b^	71.8 (65.6–78.0)^c^
total protein (g/dl)	6.48 (6.46–6.52)	6.48 (6.33–6.63)	6.66 (6.55–6.78)^c^
serum albumine (g/dl)	3.78 (3.76–3.80)	3.74 (3.63–3.84)	3.50 (3.42–3.58)^c^
serum creatinine (mg/dl)	11.2 (11.1–11.4)	10.7 (9.94–11.5)	10.4 (9.87–11.0)^b^
hemoglobin (g/dl)	10.2 (10.1–10.3)	9.9 (9.50–10.4)	10.2 (9.93–10.5)
platelet count (10^4^/µl)	18.7 (18.2–19.1)	15.0 (13.0–17.0)^c^	16.1 (14.5–17.6)^c^
white blood count (/µl)	5814 (5706–5923)	5025 (4487–5562)^b^	5120 (4718–5522)^c^
hsCRP^a^ (mg/l)	1.16 (1.06–1.28)	1.40 (0.89–2.20)	1.20 (0.85–1.69)

Causes of renal filure, comorbid conditions and habits expressed as numbers (%)			
CGN	276 (28.5%)	18 (46.2%)	20 (28.6%)
DMN	241 (24.9%)	5 (12.8%)	16 (22.9%)
HTN	97 (10.0%)	4 (10.3%)	9 (12.9%)
PCK	37 (3.8%)	1 (2.6%)	0 (0.0%)
Lupus N	4 (0.4%)	0 (0.0%)	0 (0.0%)
Others	62 (6.4%)	4 (10.3%)	6 (8.6%)
unknown	251 (25.9%)	7 (17.9%)	19 (27.1%)
MI	42 (4.3%)	1 (2.6%)	1 (1.4%)
stroke	159 (16.4%)	5 (12.8%)	9 (12.9%)
malignancy	67 (6.9%)	6 (15.4%)	6 (8.6%)
DM	289 (29.9%)	5 (12.8%)	19 (27.1%)
dyslipidemia	440 (45.5%)	19 (48.7%)	32 (45.7%)
current smoker	254 (26.2%)	12 (30.8%)	28 (40.0%)^d^
past smoker	247 (25.5%)	8 (20.5%)	16 (22.9%)
regular drinker	71 (7.3%)	2 (5.1%)	2 (2.9%)

^a^hsCRP levels are expressed as adjusted geometric means (95% CIs) estimated by ANCOVA.

^b^*P* < 0.05 ^c^*P* < 0.01 estimated by multiple comparisons using Bonferroni adjustment.

^d^*P* < 0.05 estimated by chi squared test.

Abbreviations: SD, standard deviation; HD, hemodialysis; CI, confidence interval; SBP, systolic blood pressure; HDLC, high-density lipoprotein cholesterol level; LDLC, low-density lipoprotein cholesterol level; hsCRP, serum high-sensitive C reactive protein; CGN, chronic glommerulonephritis; DMN, diabetic nephropathy; HTN, hypertensive nephrosclerosis; PCK, congenital polycystic kidney disease; Lupus N, lupus nephritis; MI, myocardial infarction; DM, diabetes mellitus; ANCOVA, analysis of covariance.

The proportion of current smokers in group C was the highest of the 3 groups (χ^2^ = 6.47, *P* = 0.03). There was no significant difference among groups in the proportions of patients with chronic glomerulonephritis (χ^2^ = 5.66, *P* = 0.06), diabetic nephropathy (χ^2^ = 3.06, *P* = 0.22), diabetes mellitus (χ^2^ = 5.41, *P* = 0.07), or past histories of myocardial infarction (χ^2^ = 1.65, *P* = 0.44), stroke (χ^2^ = 0.93, *P* = 0.63), or malignancy (χ^2^ = 4.12, *P* = 0.13). There were 4233 observed patient-years, after 5 years of follow-up. The mean and median follow-up periods were 3.9 and 4.9 years, respectively. A total of 406 patients died during the 5-year observation period.

**Figure 3.**
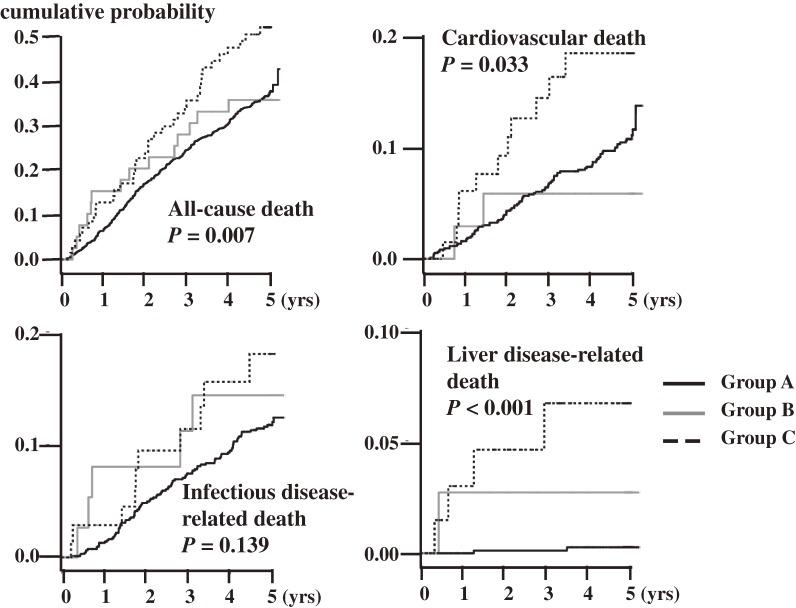
Estimated Kaplan-Meier cumulative probability of death in the 3 groups. The upper left graph shows the cumulative probability of all-cause death. Patients in group C had a significantly higher probability of death than did patients in group A (*P* = 0.007, log rank test), but there was no significant difference in probability of death between groups A and B (*P* = 0.174). The upper right graph shows the cumulative probability of cardiovascular death. Patients in group C had a significantly higher probability of cardiovascular death than did patients in group A (*P* = 0.033); the probability of cardiovascular death did not significantly differ between groups A and B (*P* = 0.118). The lower left graph shows the cumulative probability of infectious disease-related death, which did not significantly differ among the 3 groups. The lower right graph shows the cumulative probability of liver disease-related death. Patients in group C had a significantly higher probability of liver disease-related death as compared with patients without HCV infection (*P* < 0.001). The probability of liver disease-related death in group B did not significantly differ from that of group A (*P* = 0.457).

Figure 3 shows the Kaplan-Meier estimated cumulative probabilities of death for patients in the 3 groups. The cumulative probability of all-cause death (upper left) in group C was significantly higher as compared with group A (*P* = 0.007, log rank test), but not as compared with group B (*P* = 0.174). Group C also had higher probabilities of cardiovascular death (upper right, *P* = 0.033) and liver disease-related death (lower right, *P* < 0.001) as compared with group A. Group B did not have significantly higher probabilities of cardiovascular death (*P* = 0.118), infectious disease-related death (*P* = 0.775), or liver disease-related death (*P* = 0.457).

Table 3 shows the number of deaths, crude mortality rates, and sex- and age-adjusted mortality rates per 1000 patient-years (95% CIs), and relative risks for death expressed as sex- and age-adjusted relative mortality rate ratios (95% CIs) in group B and group C as compared with the reference (group A). The crude mortality rates in groups A, B, and C were 92.7, 94.0, and 147, respectively. Sex- and age-adjusted mortality rates (95% CI) in groups A, B, and C were 71.9 (62.6 to 81.3), 80.4 (37.9 to 123), and 156 (104 to 207), respectively. The relative risks (95% CI) for all-cause, cardiovascular, infectious disease-related, and liver disease-related death in group C were 2.16 (1.53 to 3.07), 1.98 (1.19 to 3.28), 2.46 (1.27 to 4.76), and 30.8 (5.34 to 178), respectively. In contrast, group B did not have significantly higher risks for death, with the exception of liver disease-related death (RR, 13.7; 95% CI, 1.24 to 152).

**Table 3.** Number of deaths, crude and sex- and age-adjusted mortality rates, and relative risks (RRs) for death compared with references by groups according to HCV seropositivity

HCV seropositivity status groups (number of subjects)	group A HCV Ab(−) *n* = 968	group B and C HCV Ab(+) *n* = 109	group B	group C	All subjects *n* = 1077
	
			HCV Ab(+) Ag(−) *n* = 39	HCV Ab(+) Ag(+) *n* = 70	
all-cause death (crude mortality)	356 (92.7)	50 (127)	14 (94.0)	36 (147)	406 (95.9)
adjusted mortality (95% CI)	71.9 (62.6–81.3)	123 (88.6–158)	80.4 (37.9–123)	156 (104–207)	96.5 (75.5–118)
RR (95% CI)	REF	1.71 (1.27–2.30)	1.12 (0.66–1.91)	2.16 (1.53–3.07)	

cardiovascular (crude mortality)	173 (45.1)	21 (53.4)	4 (26.9)	17 (69.5)	194 (45.8)
adjusted mortality (95% CI)	37.2 (30.5–43.9)	53.0 (30.0–76.0)	24.3 (0.40–48.2)	73.5 (38.0–109)	40.5 (25.3–55.7)
RR (95% CI)	REF	1.42 (0.90–2.44)	0.65 (0.24–1.76)	1.98 (1.19–3.28)	

infectious disease-related (crude mortality)	97 (25.3)	15 (38.1)	5 (33.6)	10 (40.9)	112 (26.5)
adjusted mortality (95% CI)	17.8 (13.1–22.5)	35.6 (17.1–54.1)	25.7 (2.70–48.7)	43.6 (16.3–71.0)	27.1 (16.5–37.7)
RR (95% CI)	REF	1.99 (1.15–3.43)	1.45 (0.50–3.56)	2.46 (1.27–4.76)	

liver disease-related (crude mortality)	2 (0.52)	5 (12.7)	1 (6.71)	4 (16.4)	7 (1.65)
adjusted mortality (95% CI)	0.40 (0.00–1.10)	9.80 (0.00–21.5)	5.70 (0.00–17.4)	12.8 (0.00–29.6)	3.10 (0.00–6.60)
RR (95% CI)	REF	24.2 (4.56–128)	13.7 (1.24–152)	30.8 (5.34–178)	

Crude and sex- and age-adjusted mortality rates (95% confidence intervals) are expressed as /1000 person-years. Adjusted mortalities and relative risks (expressed as relative mortality rate ratios) were estimated by Poisson regression analysis.

Table 4 shows the relative risks for each cause of death in groups B and C, as compared with the reference (group A), expressed as multivariate-adjusted mortality rate ratios. The RRs for all-cause and cardiovascular death in group C were approximately 2.0, which indicated significantly higher risks for such deaths in group C. The RR for infectious disease-related death in group C was also approximately 2.0, although the result was of marginal significance (*P* = 0.051 after model A adjustment; *P* = 0.14 after model B adjustment). In contrast, the RRs for all-cause, cardiovascular, and infectious disease-related death in group B ranged from 0.75 to 1.66, and there was no significant increase in the risk of such deaths. The risk of liver disease-related death was 15.3 in group B and 28.8 in group C, which were significantly higher as compared with the reference group.

**Table 4.** Relative risks (RRs) for each cause of death compared with references by groups according to HCV seropositivity

HCV seropositivity status groups	group A HCV Ab(−)	group B and C HCV Ab(+)	group B	group C
	
			HCV Ab(+) Ag(−)	HCV Ab(+) Ag(+)
all-cause death				
model A	Ref	1.62 (1.20–2.18)	1.24 (0.72–2.12)	1.83 (1.29–2.59)
model B		1.48 (1.09–2.00)	1.23 (0.72–2.12)	1.60 (1.13–2.28)

cardiovascular death				
model A	Ref	1.42 (0.89–2.24)	0.75 (0.28–2.04)	1.79 (1.08–2.97)
model B		1.33 (0.84–2.11)	0.75 (0.28–2.02)	1.64 (0.98–2.73)

infectious disease-related death				
model A	Ref	1.83 (1.05–3.19)	1.66 (0.66–4.13)	1.94 (0.99–3.75)
model B		1.60 (0.91–2.80)	1.64 (0.65–4.15)	1.58 (0.81–3.07)

liver disease-related death				
model A	Ref	18.6 (3.51–98.1)	8.55 (0.75–98.1)	26.6 (4.57–155)
model B		22.9 (3.53–149)	15.3 (1.26–186)	28.8 (3.75–221)

Crude and adjusted mortalities (95% confidence intervals) are expressed as /1000 person-years.

Adjusted mortalities were estimated by Poisson regression analysis after adjusting for risk factors.

Adjustment for risk factors

model A: age, male gender, high BMI, dyslipidemia, diabetes, high blood pressure, history of myocardial infarction, stroke, or malignant diseases, smoking habit and regular drinking habit.

model B: model A plus low BMI, low blood pressure, high CRP level, and hypoalbuminemia.

## DISCUSSION

In this study, we estimated crude and sex- and age-adjusted rates for all-cause death and cause-specific death in hemodialysis patients who were negative for HCV antibodies, those who were positive for HCV antibodies, and those who were positive for both HCV antibodies and anti-HCV core antigen antibodies. We also calculated the relative risks of all-cause death and cause-specific death in patients positive for HCV antibodies only and patients positive for both HCV antibodies and anti-HCV core antigen antibodies as compared with patients who were negative for anti-HCV antibodies. These 3 groups roughly correspond to patients without HCV infection (group A), patients with past HCV infection (group B), and patients with chronic HCV infection (group C). Therefore, the results showed higher risks of all-cause, cardiovascular, infectious disease-related, and liver disease-related death among the chronic HCV subgroup, whereas past HCV infection was not associated with increased risk of any cause of death, except liver disease-related death.

Most prior studies investigated only the relative risks of all-cause and/or cause-specific death attributable to HCV infection among hemodialysis patients, without further differentiating between past and chronic HCV infection.^19^^,^^22^^,^^24^^,^^25^ In a meta-analysis, Fabrizi et al found that the adjusted RR for all-cause mortality due to HCV infection was 1.34.^27^ However, Stehman-Breen et al used quantitative estimation of HCV RNA levels to determine whether chronic HCV infection increased mortality among hemodialysis patients: the multivariate-adjusted RR for all-cause death attributable to chronic HCV infection was 2.0.^23^ Other studies reported a multivariate-adjusted RR of death attributable to HCV infection (including past HCV infection) between 1.2 and 1.6.^27^ The lower relative risks in studies that assessed HCV status using antibody-based techniques may be due to underestimation related to the inclusion of patients with past HCV infection only. In our study, the RR of all-cause death due to chronic HCV infection, as determined by quantitative estimation of HCV core antigen, was 1.83 after traditional risk factor adjustment, which is similar to the RR of 2.0 reported by Stehman-Breen et al. Taken together, both previous studies and the present study suggest that it is mainly chronic HCV infection that increases the risks of all-cause and cause-specific death.

The causes of death that contribute to increased mortality among hemodialysis patients with chronic HCV infection were not fully identified in previous studies. In a meta-analysis, Fabrizi et al showed that HCV-seropositive hemodialysis patients had higher rates of liver disease-related death than their seronegative counterparts, but that cardiovascular and infectious disease-related morality rates were similar.^27^ The studies included in their meta-analysis all cited cardiovascular death as the most common cause of death in dialysis patients. Excess deaths attributable to HCV infection cannot be explained by an increase in the number of HCV-attributable liver disease-related deaths. Whether cardiovascular death (the most common cause of death) and infectious disease-related death (the second most common cause of death) increase mortality among hemodialysis patients is also important, as is the contribution of liver disease-related death.

We are unable to explain the increased risks of cardiovascular death and infectious disease-related death among hemodialysis patients who were positive for anti-HCV core antigen antibodies in the present study. Cross-sectional analysis of baseline data provides some clues regarding possible mechanisms that might explain the association between anti-HCV core antigen positivity and increased cardiovascular and infectious disease-related mortality risk. Despite being younger, patients who were positive on the anti-HCV core antigen test had lower levels of serum lipids and albumin as compared with patients who were negative on the HCV antibody test. These findings suggest that hemodialysis patients who were positive for anti-HCV core antigen antibodies had hypocholesterolemia and hypoalbuminemia. Thus, insufficient levels of serum cholesterol and albumin might be associated with a malnutrition-inflammation syndrome activated by chronic HCV infection. Such a syndrome might lead to immune dysfunction, resulting in an increased risk of cardiovascular and infectious disease-related death.^19^^–^^31^

Several limitations in our study should be noted. Because we enrolled only 70 patients who were positive for anti-HCV core antigen, we were not able to perform an accurate sex-stratified risk assessment of cause-specific death. Sex differences in the risk of each cause of death might exist, and the relationships should therefore be re-examined in larger cohort studies or in meta-analyses using data from patients whose chronic HCV infection status is precisely defined. Lack of high-sensitivity, quantitative HCV-RNA data from patients who were positive for HCV antibody and negative for HCV core antigen is a major limitation of this study. It is possible that hemodialysis patients who are negative for HCV core antigen nevertheless have very low levels of HCV-RNA; however, the possibility of missing such cases is very low because none of the population-based controls in our previous study were simultaneously positive for HCV-RNA and HCV antibody and negative for HCV core antigen.^21^ Therefore, we believe that the results of the current study are not distorted by the lack of HCV-RNA data.

Because second-generation ELISA became available as a clinical diagnostic tool in 1992, patients who began hemodialysis treatment before 1992 might have had more exposures to infection and a higher incidence of HCV infection. It remains to be clarified whether HCV infection, and a long history of hemodialysis treatment, independently increase the risk of death. In a separate analysis, we estimated the risk of each cause of death attributable to HCV infection only in patients who started hemodialysis treatment after 1992. The results were similar to those from analyses of all subjects (data not shown).

We determined HCV infection status based on baseline information only. Changes in HCV infection status (eg, incident HCV infection during the observation period) were not considered. Previous studies showed that the incidence of HCV infection in hemodialysis patients was lower than 0.5% per year.^11^^,^^32^ The risk of death attributable to HCV infection may have been underestimated, and putative underestimation of relative risks of death is not negligible.

Despite its limitations, our simple and economic method of determining HCV infection status provided sufficient results to discern a difference in mortality between patients with past versus chronic HCV infection. Furthermore, limiting the analysis to patients with chronic HCV infection enabled us to show that an increased risk of death among hemodialysis patients with HCV infection was due to an increased risk of cardiovascular and infectious disease-related deaths, as well as the increased risk of liver disease-related death. We conclude that more attention should be paid to chronic HCV infection in hemodialysis patients.
